# AxonPacking: An Open-Source Software to Simulate Arrangements of Axons in White Matter

**DOI:** 10.3389/fninf.2017.00005

**Published:** 2017-01-31

**Authors:** Tom Mingasson, Tanguy Duval, Nikola Stikov, Julien Cohen-Adad

**Affiliations:** ^1^NeuroPoly Lab, Institute of Biomedical Engineering, Polytechnique MontrealMontreal, QC, Canada; ^2^Signal Processing Department, École Centrale de NantesNantes, France; ^3^Department of Biomedical Engineering, Montreal Heart Institute, University of MontrealMontreal, QC, Canada; ^4^Functional Neuroimaging Unit, Centre de Recherche de l'Institut Universitaire de Gériatrie de Montréal (CRIUGM), Université de MontréalMontreal, QC, Canada

**Keywords:** quantitative MRI, myelin volume, fraction restricted, axon density, disks packing, white matter, microstructure

## Abstract

**HIGHLIGHTS**

- AxonPacking: Open-source software for simulating white matter microstructure.
- Validation on a theoretical disk packing problem.
- Reproducible and stable for various densities and diameter distributions.
- Can be used to study interplay between myelin/fiber density and restricted fraction.

Quantitative Magnetic Resonance Imaging (MRI) can provide parameters that describe white matter microstructure, such as the fiber volume fraction (FVF), the myelin volume fraction (MVF) or the axon volume fraction (AVF) via the fraction of restricted water (*fr*). While already being used for clinical application, the complex interplay between these parameters requires thorough validation via simulations. These simulations required a realistic, controlled and adaptable model of the white matter axons with the surrounding myelin sheath. While there already exist useful algorithms to perform this task, none of them combine optimisation of axon packing, presence of myelin sheath and availability as free and open source software. Here, we introduce a novel disk packing algorithm that addresses these issues. The performance of the algorithm is tested in term of reproducibility over 50 runs, resulting density, and stability over iterations. This tool was then used to derive multiple values of FVF and to study the impact of this parameter on *fr* and MVF in light of the known microstructure based on histology sample. The standard deviation of the axon density over runs was lower than 10^−3^ and the expected hexagonal packing for monodisperse disks was obtained with a density close to the optimal density (obtained: 0.892, theoretical: 0.907). Using an FVF ranging within [0.58, 0.82] and a mean inter-axon gap ranging within [0.1, 1.1] μm, MVF ranged within [0.32, 0.44] and *fr* ranged within [0.39, 0.71], which is consistent with the histology. The proposed algorithm is implemented in the open-source software AxonPacking (https://github.com/neuropoly/axonpacking) and can be useful for validating diffusion models as well as for enabling researchers to study the interplay between microstructure parameters when evaluating qMRI methods.

## Introduction

The white matter contains bundles of myelinated nerve cell projections (axons). Over the past years, Magnetic Resonance Imaging (MRI) has seen the development of quantitative metrics that can provide microstructural information about these axons, such as the myelin volume fraction (MVF), the intra-axonal volume fraction via the fraction of restricted water (*fr*), and the ratio of the inner to the outer diameter of the neuronal fibers (g-ratio) in white matter (Stanisz et al., 1997; Laule et al., 2007; Assaf et al., 2008; Fieremans et al., 2008, 2016; Alexander et al., 2010; Stikov et al., 2011). However, the realistic ranges for these parameters, as well as their sensitivity to microstructural variation (e.g., changes in axon density, axon diameter distribution, g-ratio) are not clear. While some relationships can be derived using analytical equations (e.g., myelin content can be related to fiber volume fraction (FVF), assuming g-ratio is fixed), other mathematically complex relationships, such as the axon density as a function of axon diameter distribution, requires simulations.

Assuming parallel fibers, which is typical for model-based quantitative diffusion metrics (Assaf et al., 2008), the geometrical simulation of a fiber bundle can be reduced to a two-dimensional polydisperse disks packing problem. Despite the presence of neurite orientation dispersion (Ronen et al., 2014), the 2D reduction can be useful and is already a close representation of what is observed in histology in the case of quasi-parallel fibers (Zaimi et al., 2016). Particle (disks or spheres) packing has been extensively studied in the past decades with a wide spectrum of applications in the fields of physics, industry and mathematics. Examples of applications are the modeling of granular media (Zhang and Makse, 2005; Isola, 2008) or powder and fluid (Bernal and Mason, 1960; Yu et al., 1997; Williams and Philipse, 2003), optimal arrangement of cylindrical products in a container (Dowsland, 1991) or electrical wires in a bundle (Sugihara et al., 2004) or lastly conformal mapping on a surface (Collins and Stephenson, 2003).

While particle packing is a very complex optimization problem (Lenstra and Rinnooy, 1979), multiple approaches exist.

Unfortunately, most of these algorithms were developed with specific constraints in mind that are not suitable for white matter. Many algorithms place each particle (disk or sphere) within a container which limits the maximal disk density and usually requires strategies that change the diameter distribution or place same-sized particles close together in the packing (George et al., 1995; Graham et al., 1998; Wang et al., 2002; Gensane, 2004; Stoyan and Yaskov, 2008; Belevičius et al., 2011).

In the case of polydisperse disk simulations without border constraints, a first approach consists of representing a fixed graph of disk centers positions and then finding the configurations of each individual disk diameter satisfying preassigned patterns of tangency (Collins and Stephenson, 2003). A second approach is to initialize the packing randomly (with a condition to prevent overlapped disks) and fill the empty spaces with smaller disks (Bagi, 2005). However, these two approaches cannot build packing from a set of particles whose sizes are user-defined, and also they do not guarantee maximum packing.

Algorithms that are free from the above-mentioned constraints can be found in the field of particle dynamics (in solid, fluid, or gas). Two main methods exist for such simulations: (i) dynamic approaches (molecular dynamic) where the disks are hard or soft particles obeying mechanical and energetic conservation laws (Donev et al., 2005) and (ii) non-deterministic approaches based on Boltzmann probabilities (Frenkel and Smit, 2006). In molecular dynamics (MD) particles can be displaced either synchronously in small time steps and detoured when overlap occurs (time-driven MD), or according to a list of events, such as collisions between particles ordered in time (event-driven MD). An event-driven based packing algorithm was presented by Donev et al^1^ in which disks collide and expand uniformly until a jammed state is reached. They showed that this jammed state, called the maximally random jammed (MRJ) state, is different (lower density) than the optimal density packing, called the random close packing (RCP) (Torquato, 2013). Interestingly, the MRJ state is dependent on the algorithm used (Torquato, 2013) which mean that final packing density depends on the way disks are packed, stirred and shaken (making difficult the comparison of performance between different algorithms). The event-driven based approach is more mathematically rigorous and prevents overlapping. This algorithm requires hard wall in order to achieve a jammed state. One drawback of the event-driven approach is that it is more difficult to adapt to arbitrary shapes (i.e., other than disks). Also, this algorithm does not allow to have gaps between disks and it was not designed to simulate the white matter, therefore useful outputs are not available (e.g., MVF, FVF).

Regarding the simulation of white matter axons specifically, several simulators have also been proposed. In order to reach high density, Hall and Alexander^2^ (Hall and Alexander, 2009) used an iterative diameter increase approach in which disks can be removed to avoid overlap and boundary effects. As a consequence the final density is neither optimal nor reproducible, and it cannot be set by the user. An advantage of CAMINO is that edge effects are addressed by reproducing the pattern at the four edges of the image, which can be useful for running Monte Carlo simulations on small substrates. An alternative method for disk packing has recently been proposed (Mesri et al., 2016), which places axons iteratively in a controlled manner in order to achieve maximal density. However, this algorithm is not publicly available. Dougherty and Sveinsson, 2011^3^ used a triangle mesh to determine the position of the axons, and while this simulator has the advantage of being 3D, the fiber density cannot easily be maximized. Balls and Frank (2009) proposed to add fibers across several iterations until a user-defined density is reached, which precludes the possibility to optimize packing from it. When looking at the problem in 3D, non-parallel fibers are usually simulated randomly without packing optimization (Kamiya et al., 2015).

In this paper, we present AxonPacking^4^, a novel, easy-to-use and open-source Matlab algorithm for simulating axon packing with user-defined diameter distribution of the axons and the gap between axons. This algorithm is based on (MD)without any border constraints. Disk (i.e., axon) density is optimized by migrating disks toward the center of the 2D space. A couple of features were specifically added to model the white matter realistically: gamma distributions of axon diameter, presence of a gap between axons, and myelin thickness. Also a couple of outputs, related to quantitative MRI metrics, were added, such as the (MVF), (FVF) (i.e., disk density), and the (fr). This simulator was used to extract realistic ranges of MVF and fr.

## Methods

### White matter model

White matter tissue is divided in three compartments: axons, myelin sheath and extra-axonal space. Axons are assumed to be parallel cylinders, therefore the invariance along the fiber axis makes it possible to consider this problem in 2D. The assumption of parallel fibers is adapted for regions presenting a good coherence of orientation of the neuronal bundles, such as in the spinal cord. The dense packing of axons is thus equivalent to the generation of random 2-dimensional packing of N perfectly round and non-compressible disks. As shown in previous histological studies (Pajevic and Basser, 2013; Sepehrband et al., 2016), axon diameter distributions follow a Gamma distribution (defined by its mean μ and variance σ^2^). The Gamma probability for a diameter d is defined as follows (with Γ the Gamma function of Euler): $Prob_{Gamma}\left(d,\;\mu ,\;\sigma \right)\;=\;d^{a-1}*exp\left(-\frac{d}{b}\right)\;/\;\left(\Gamma \left(a\right)\;*\;b^{a}\right)\;where\;a={\left(\mu /\sigma \right)}^{2}$ and *b* = σ^2^/μ.

**Figure 1:**
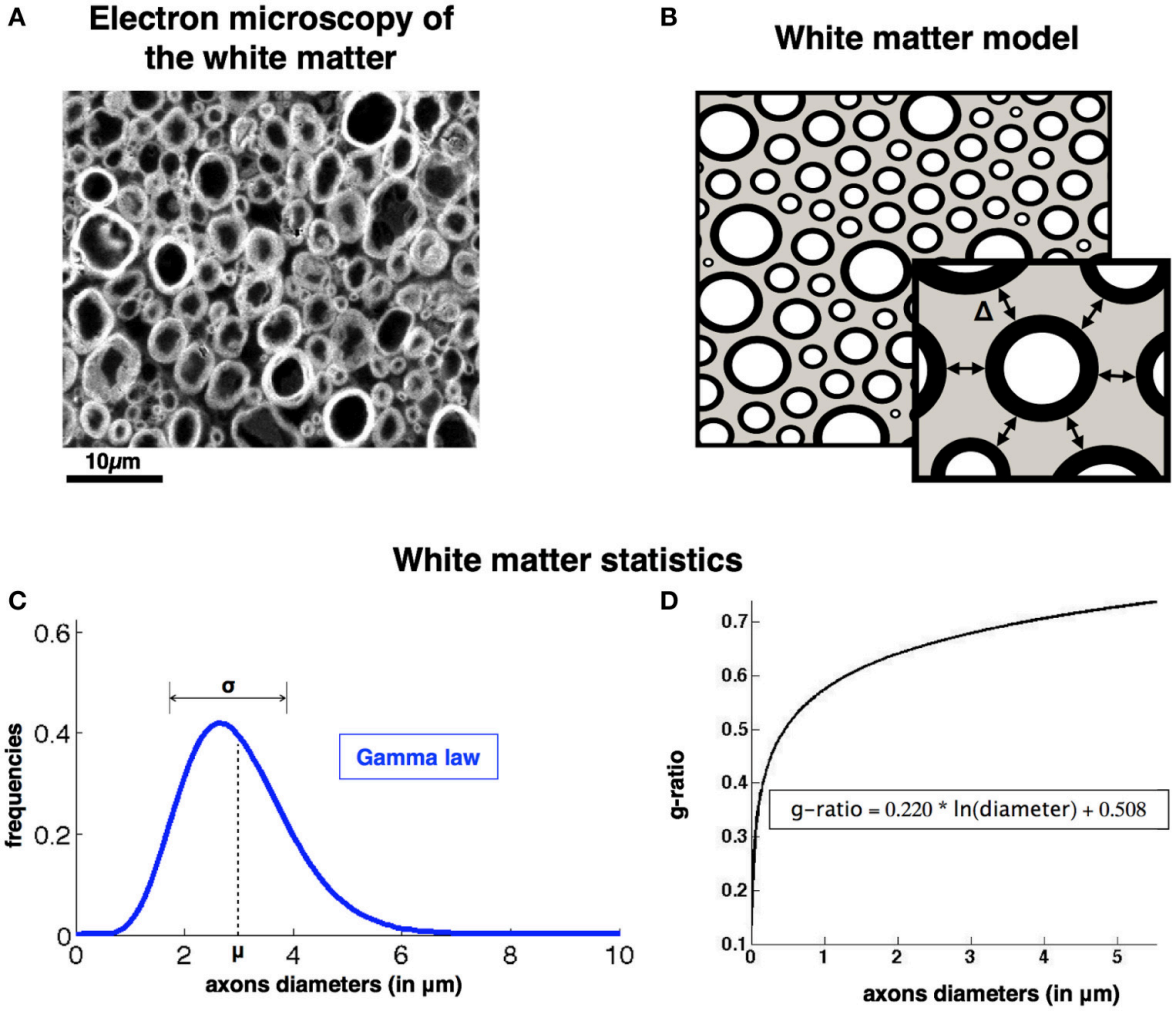
**AxonPacking model (A)**. Electron microscopy of a ventral region of a rat spinal cord stained with osmium. Since most fibers are running along the spinal cord, axons appear like densely packed disks. Three regions can be identified: gracilis (blue), cuneatus (red) and lateral corticospinal (green). **(B)**. Corresponding Model of the white matter with three compartments: extra-axonal space (gray), myelin sheath (black), intra-axonal (white). Graphical illustration of the gap between axons Δ: the periphery of the center disk is at a distance Δ from the periphery of the other six disks in its neighborhood. **(C)**. Axon diameter distribution is modeled by a Gamma law described by the mean (μ) and standard deviation (σ). **(D)**. Relationship between the g-ratio and the axon diameters.

Axons are spaced from each other by a gap Δ (see illustration in Figure 1). This gap is assumed constant in our model and is not optimized along the packing process. Axons are surrounded by a myelin sheath. The ratio of the inner (*d_unmyelinated_*) to the outer diameter (*d_myelinated_*) of this myelin sheath is called the fiber g-ratio: *g*_*ratio*_ = *d*_*unmyelinated*_ / *d*_*myelinated*_. Interestingly the g-ratio is fairly constant across species and white matter regions (Rushton, 1951; Chomiak and Hu, 2009) and is dependent mostly on the diameter of the axon according to the relationship presented in Ikeda and Oka (2012): *g_ratio_* = 0.220 * *log*(*d_unmyelinated_*) + 0.508 (see the plot on Figure 1). Note that this relation was derived from axons of the peripheral nervous system, but similar trends can be observed in the central nervous system (West et al., 2015). See discussion (section Using AxonPacking for Modeling White Matter) for more details.

### Axonpacking algorithm

**Figure 2:**
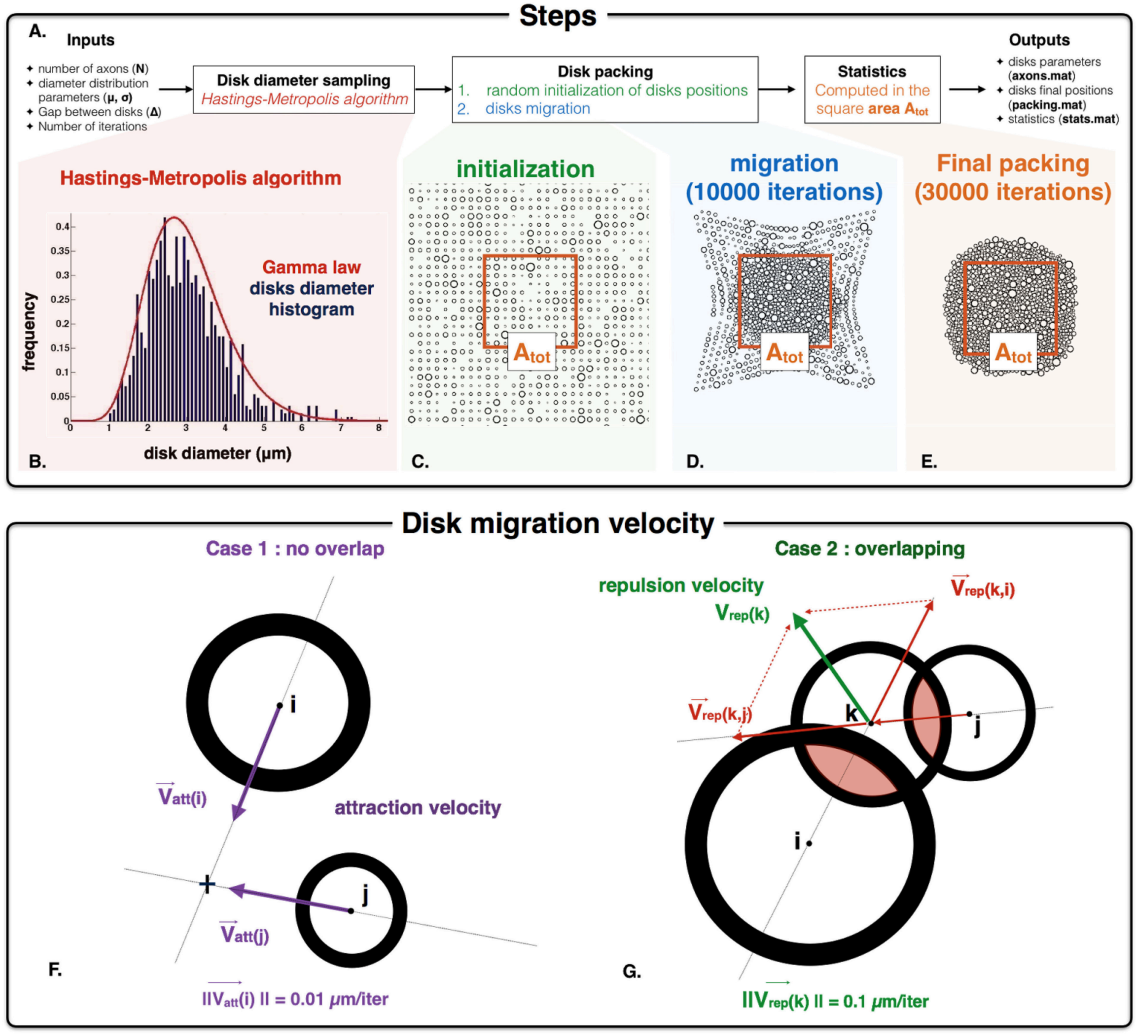
**AxonPacking algorithm (A)**. Overall procedure for disk packing algorithm. **(B)** Histogram distribution of disk diameter. In this example, *N* = 1000 diameters are simulated, and the desire Gamma distribution of disks diameter is μ = 3 μm and σ^2^ = 1 μm (red curve). Our implementation of the Hasting Metropolis algorithm generate disks diameter (blue histogram) that correctly match this distribution. **(C)** Initialization of the disks randomly chosen on a grid. The area A_*tot*_ defines the area where the density FVF is computed (used to control the convergence of AxonPacking). **(D)** Migration of the disks toward the center. **(E)** Final packing where all the statistics are computed. **(F,G)**. Graphical illustration of the attraction velocity that operates for every non-overlapping disks **(F)** and the repulsion velocity for the overlapping ones **(G)**.

The simulator is implemented in Matlab (R2014a). The different steps to process packing are the following (Figure 2A): (i) the diameters of the disks are randomly chosen using a Gamma distribution parameterized with the mean (μ), standard deviation (σ) and number of axons (N); (ii) the positions of disks are initialized on a grid, and then migrate toward the center of the packing area until the maximum disk density is achieved. A video illustrating the packing is available at: http://www.neuro.polymtl.ca/downloads. The random disk diameters are obtained using a Hasting Metropolis algorithm (Chib and Greenberg, 1995). Briefly, from an initial diameter value, subsequent diameters are randomly drawn with a Gaussian law and accepted (or rejected) according to a probability depending on the Gamma probability density function. The acceptance probability over and under two fixed diameter values is forced to zero. These two thresholds (set to 0.2 and 10 μm by default) avoid unrealistically small or large axons. Figure 2B shows an example of sampling for *N* = 1000 disks, μ = 3 and σ^2^ = 1 μm. From the set of N disks following the expected diameter distribution, disk positions are initialized and then move along the iteration packing process according to specific rules. To account for the gap Δ between the disks during the packing process, disks are considered having a diameter d' = d+Δ. The main inputs and outputs of the algorithm are the following:

**Inputs**: 5 Variables

- The number of disks i.e., axons to include in the simulation (*N* in AxonPacking),
- The diameter distribution parameters: mean μ and variance σ^2^ of disk diameters (*d_mean* and *d_var* in AxonPacking),
- The fixed gap between the edge of disks Δ (*Delta* in AxonPacking),
- The number of iterations i.e., disk migrations performed by the algorithm before computing the outputs (*iter_max* in AxonPacking).

**Outputs**: 3 Matlab structures and one image

- The axon features (*N, d_mean, d_var, Delta, g_ratio*, and the drawn diameters *d*) stored in *axons.mat*,
- The packing results (initial positions of disks (*initial_positions*) and final positions of disks (*final_positions*) stored in *packing.mat*,
- The statistics results with the values for each metric computed in the packing (*FVF, FR, MV*F, AVF) stored in stats.mat,
- A png image of the final packing with three different labels (intra-axonal, myelin and extra-axonal).

#### Initialization

The disks are randomly initialized on a grid within a square area (Figure 2C). To initialize the disk positions in such a way that the disks do not overlap and are closed enough to each other the size of the square area is set to $\sqrt{N*{\left(2*max\left(R_{k},\;k=1..N\right)+\Delta \right)}^{2}}\;$ (*R*_*k*_ the radii, Δ the gap between disks) and the spacing in height and width of the grid is set to the size of the square area divided by $\sqrt{N}+1$. In this way every disk is initialized in a square of the grid whose side is larger than the disk diameter (Figure 2C).

#### Migrations

After that, at each iteration, every single disk migrates (Figure 2D) following the conditions defined in the next paragraph. At each iteration, the velocity of each disk is computed according to two different situations. In case of the absence of overlapping with any other disk, the velocity **V**_att_ is a constant attraction toward the center of the square area (Figure 2F). Its direction is computed from the positions of the disk and the center of the square area. Its norm is fixed to 0.01 μm/iteration. At each iteration, disk overlapping condition is checked by computing the matrix **P** of distance between pairs of disk. In case of overlap (negative values in **P**), the attraction term is omitted and the velocity V_rep_ is the result of the repulsion between two disks. The velocity vector is defined by its direction and its norm. The direction of this velocity vector is defined by the center of the two disks that are in conflict. If multiple disks are in conflict, the direction for the disk k is calculated by summing the normalized individual velocities associated with the overlapping disks i and j (Figure 2G). The norm of the velocity vector is set to a constant *c*. In order to discriminate the overlapping as much as possible, ||**V**_rep_|| must be higher than ||**V**_att_|| i.e., *c* > 0.01 μm/iteration. However, ||**V**_rep_|| must be lower than the disk diameters to avoid bouncing of disks. It was found that a value of *c* = 0.1 μm/iteration is a good compromise between the repulsive and the attractive velocities in order to have (i) minimal overlapping between disks, (ii) fast convergence and (iii) no major bouncing of the disk. Note that *c* can be modified if necessary. The disk density increases over the migrations and tends toward a limit value. It is necessary to first launch the algorithm with the packing inputs (N, μ, σ, and Δ) and a high number of iterations: 35000 iterations when *N* = 1000 for example. MRI metrics, such as the disks density e.g., FVF can be calculated every *p* iterations to assess the sufficient number of iterations to reach a certain degree of precision. *p* is a user-defined integer: *p* = 250 or 1000 for example. When the packing process is finished (Figure 2E), the algorithm converts the packing image into a binary mask from which subsequent microstructure-related metrics can be derived: (FVF) e.g., the disk density, (MVF), axon volume fraction (AVF) and (fr). First a square mask is generated with three different labels (intra-axonal, myelin and extra-axonal) (see Figure 1B. for definitions) from which we compute the areas (A_*intra*−*axonal*_, A_*myelin*_ and A_*extra*−*axonal*_, respectively). This mask is located at the center of the packing and its area (A_*tot*_), and is defined such that no disks contained within the mask are located at the periphery of the packing cloud (to avoid edge effects). Note that the number of axons in the mask is consequently lower than the number of axons in the input (N). Also note that N and Δ can be fixed independently, and the resulting mask within which useful metrics are calculated (FVF, etc.) has a varying size. For each disk *i*, the intra-axonal and the myelin mask is computed using the associated g-ratio g_i_. The microstructure parameters are computed using the following formulas:

(1) $$\begin{array}{rcl} FVF & = & \frac{A_{intra-axonal}+A_{myelin}}{A_{tot}}\;MVF=\frac{A_{myelin}}{A_{tot}}\\ fr & = & \frac{A_{intra-axonal}}{A_{intra-axonal}+A_{extra-axonal}} \end{array}$$

Note that FVF, MVF and fr do not theoretically depend on the mask size, because they are normalized by the area A_*tot*_ (see section Effect of the Number of Disks).

To check the quality of the results, the overlapping area ratio R_overlap_ (= A_overlap_ / A_disks_) is reported. A_overlap_ is defined as the sum of the areas of overlap between disks, and the algorithm was designed to keep this ratio negligible (<0.1%).

### Validation and performance of axonpacking

#### Validation in theoretical packing condition

Validation of the packing algorithm was performed using the well-known problem “Hexagonal Close Packing” (HCP), which shows that the optimal solution for packing disks of the same diameter follows a hexagonal lattice structure (Chang and Wang, 2010). For such a (HCP) the disk density is: $\phi_{HCP}\;=\;\pi /\sqrt{12}≃\;0.907.$ The variance diameter σ^2^ was set to 0 and a set of *N* = 250 disks with the same diameter (4 μm) were created. The goal in this first section was to test whether the automatic packing converges toward a hexagonal structure.

In the following sections, AxonPacking was used with Gamma parameters (μ and σ) based on histology of the cervical spinal cord of cat (Zaimi et al., 2016): the mean diameter μ ranged from 2.5 to 3.5 μm and the standard deviation σ ranged from 0.5 to 2.5 μm.

#### Reproducibility over runs

Reproducibility over 50 runs was also studied for three different Gamma distributions of diameters that could be found in the spinal cord of cat: μ = 3 μm and σ^2^ = [0.5, 1.5, 2.5] μm. The following parameters were used: *N* = 1000 disks, 30000 iterations and a gap Δ = 0. Then the standard deviation of FVF across runs was studied.

#### Effect of the number of disks on the statistics

In order to test the stability of FVF, MVF and *fr* with regards to the number of axons in the area A_*tot*_, the statistics were computed with varying A_*tot*_ in a particular case: *N* = 1000, μ = 3 μm, σ^2^ = 2.5 μm, Δ = 0 and 30000 iterations. The number of disks (N_*final*_) in the area A_*tot*_ ranged from N_*final*_ = 37 (for the smallest A_*tot*_) to N_*final*_ = 888 (for the largest A_*tot*_). The maximal error on the statistic STAT was computed as follows:

$$\begin{array}{l} \left(STAT\right)=max\left(STAT\left(N_{final}>200\right)\right)-min\left(STAT\left(N_{final}>200\right)\right) \end{array}$$

#### Stability of the final solution

In order to quantify the stability of the final density in the last iterations, the density FVF was computed every 250 iterations in a particular case (*N* = 1000 axons, μ = 3 μm, σ^2^ = 1 μm, Δ = 0 and 35000 iterations is performed). Based on our preliminary experiments on several parameter sets, we observed that convergence was always reached after ~15,000 iterations. Therefore, we arbitrarily decided to evaluate the stability between 26,000 and 35,000, which we consider being a conservative range. The variation of FVF between 26,000 and 35,000 iterations was computed as follows:

$$\begin{array}{lll} \Delta \left(FVF\right) & = & max\left(FVF\left(26000\leq iteration\leq 35000\right)\right)\\ -min\left(FVF\left(26000\leq iteration\leq 35000\right)\right). \end{array}$$

### Example of application of axonpacking: dependency of fiber volume fraction and myelin content on simulator parameters μ, σ, and Δ

The algorithm was applied with features specific to the white matter in the spinal cord of cat. Packings were made for three different Gamma distributions of inner diameters. Based on the existing axon segmentation (Zaimi et al., 2016) the diameter distributions is fitted with a Gamma law for different regions of the white matter and then three pairs (μ, σ^2^) were chosen, representative of three regions of the white matter: gracilis, cuneatus and lateral corticospinal. These regions are presented **Figure 7**. For each set of axons six packings were performed with six different values for the gap: Δ = [0.1, 0.3, 0.5, 0.7, 0.9, 1.1] μm. The evolution of FVF, *fr* and MVF was assessed with respect to Δ for the three cases. N was set to 1000 and the number of iterations was set to 30000.

## Results

### Hexagonal packing in monodisperse case

**Figure 3:**
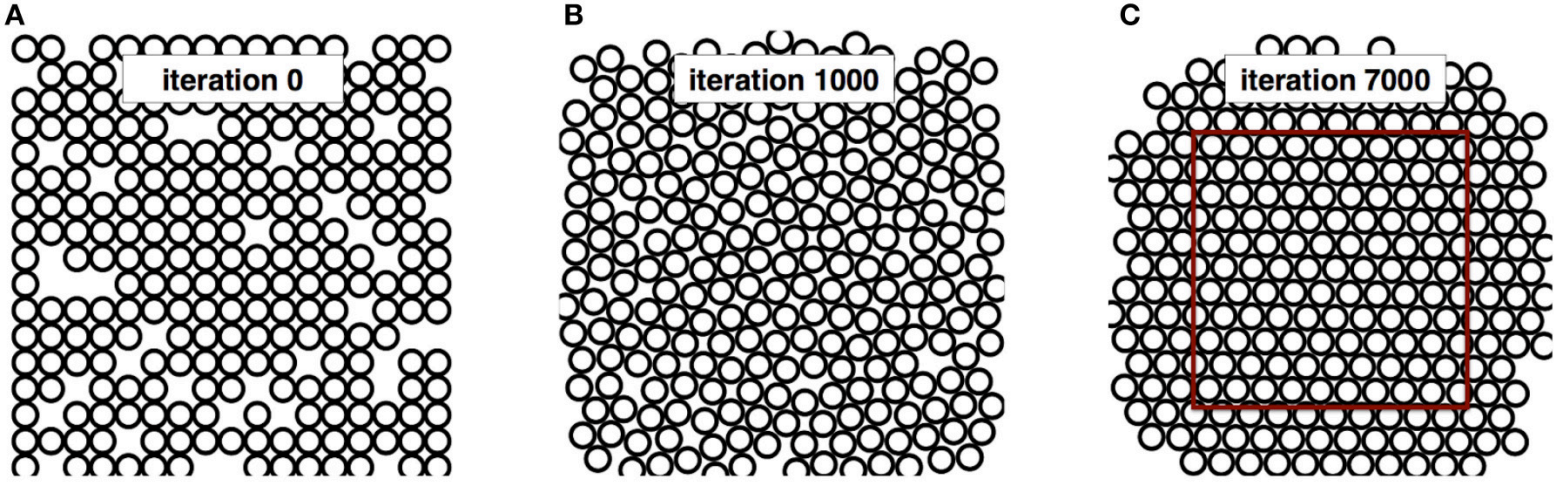
**Iteration process for the Hexagonal packing in monodisperse case (A)** initial positions of the 250 disks. **(B)** intermediate positions after 1000 migrations. **(C)** final results after 7000 migrations.

Figure 3 shows the packing at three stages of iterations: 0, 1000 and 7000. As expected from the theory, a hexagonal structure is obtained after convergence. The disk density calculated in the red rectangle for this packing after 7000 iterations is FVF = 0.892, which is close to the theoretical values FVF_HCP_ = 0.907 (2% error). The overlapping ratio is R_*overlap*_ = 0.01 %. Note that this simulation took 30 s on a iMac i5 3.4 GHz (quad core).

### Reproducibility over runs

**Figure 4:**
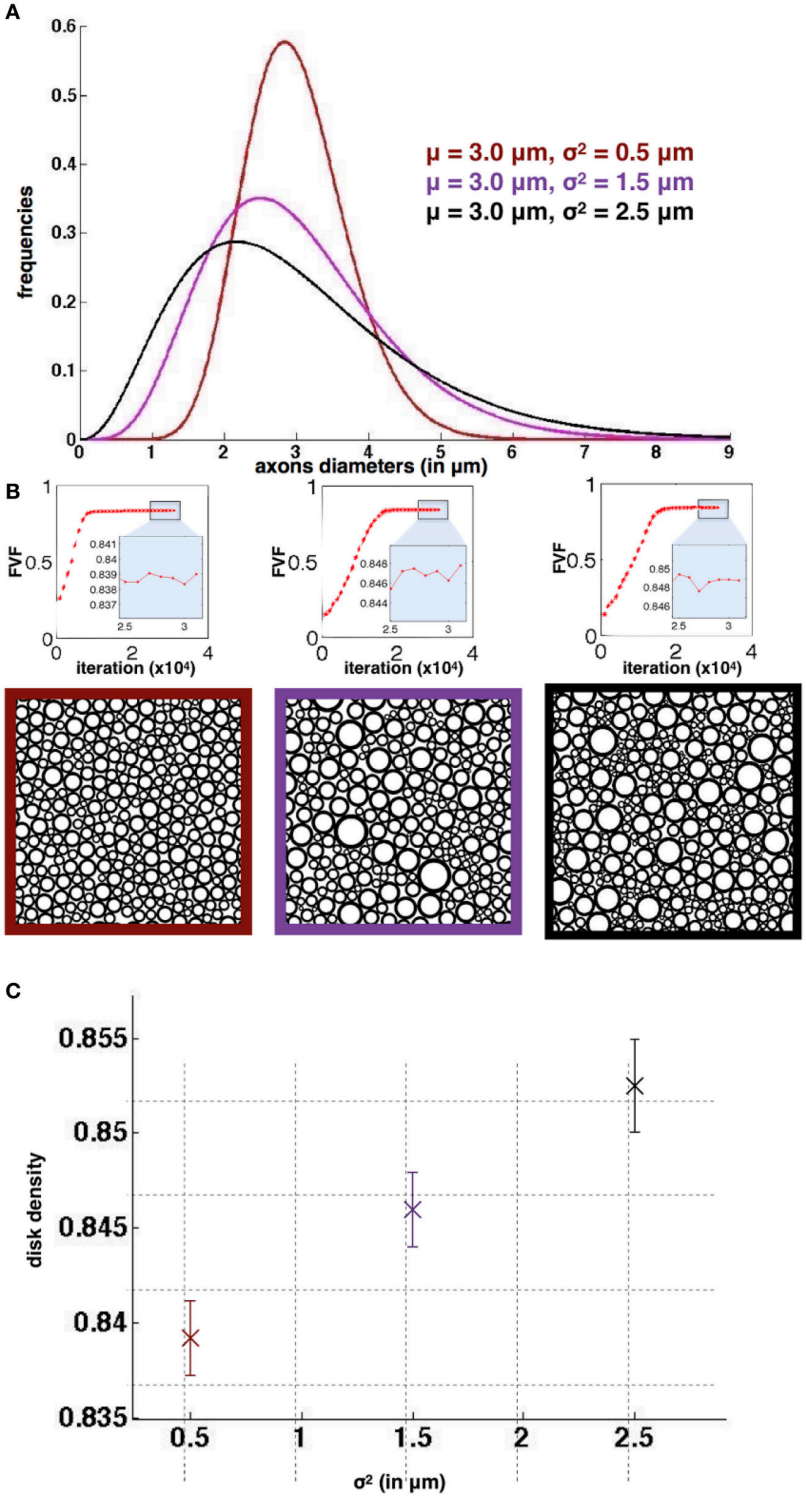
**Reproducibility of AxonPacking (A)**. Three diameters distributions. **(B)** Evolution of FVF (top) and final packing result for each distribution. The three simulations qualitatively converged before 20000 migrations. **(C)** Mean disk density (i.e., FVF) with the standard deviation over the 50 runs for the three cases.

Figure 4 shows the results of the simulation for the three different diameter distributions. The standard deviation for FVF over the 50 runs is lower than 3*10^−3^ in all 3 cases, demonstrating the good reproducibility over runs. In addition, these standard deviations are lower than the differences between the mean values (>7*10^−3^), which shows that the simulator could distinguish significantly the different cases simulated. As a result, we found an optimal density, although very close, significantly bigger for the case with larger dispersion of axon diameter. Note that R_*overlap*_ is lower than 0.1% for all packing simulations.

### Effect of the number of disks

**Figure 5:**
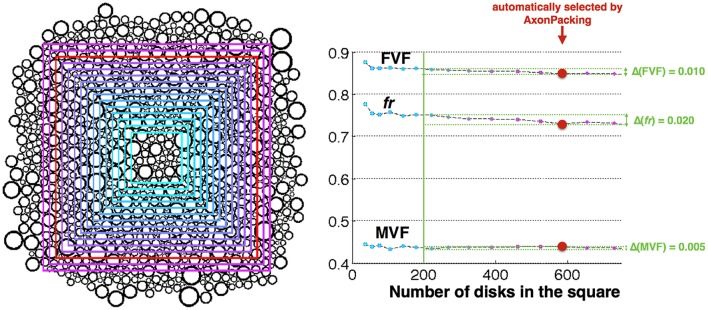
**Effect of the number of axons in the area. Left** Result of the simulation and areas A_*tot*_ where the statistics are computed. The size of the area is color-coded from cyan (smallest) to purple (biggest). The area automatically selected by AxonPacking is colored in red. **Right** FVF, fr and MVF values in the different areas (same color-coding), and measured errors (light green).

Figure 5 shows the different areas A_*tot*_, along with the FVF, MVF and *fr* values computed in these areas. For areas containing over 200 disks, FVF, MVF and *fr* were found fairly stable with an an error of 0.010, 0.020, and 0.005, respectively.

### Stability of the final solution

**Figure 6:**
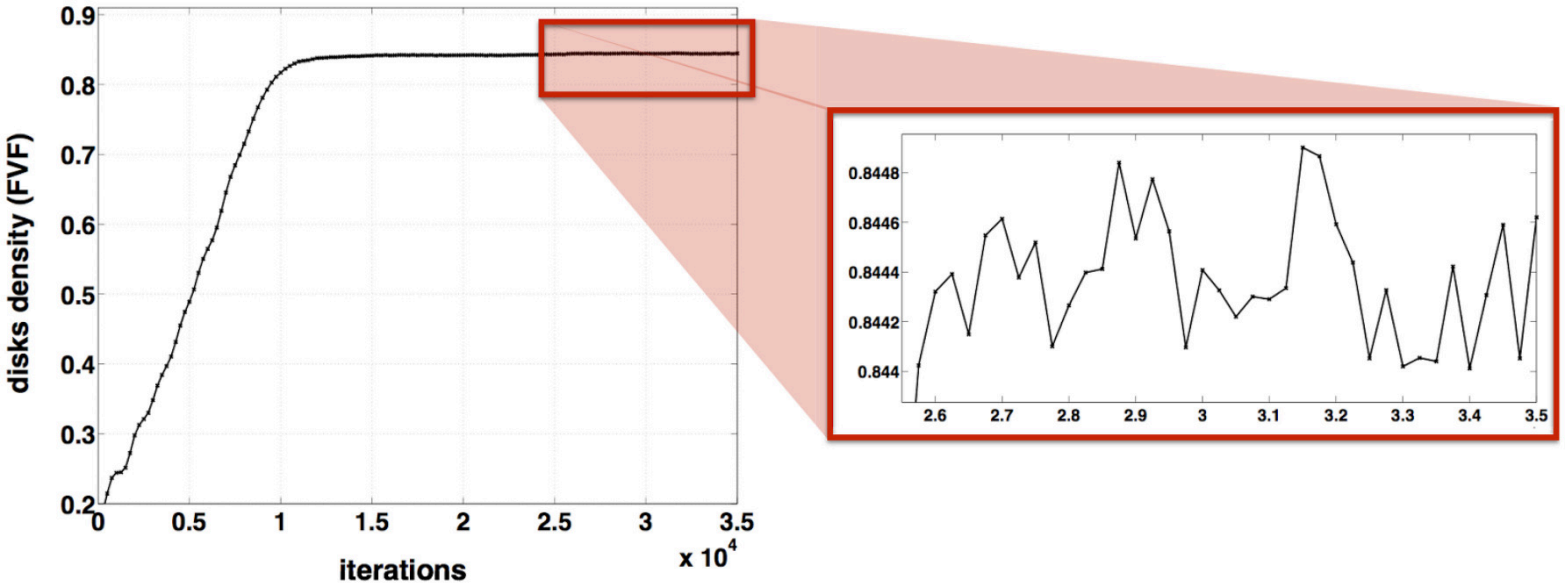
**Stability of AxonPacking**. Evolution of the disk density along the 35000 iterations for a packing of 1000 disks where μ = 3 μm, σ^2^ = 1 μm and Δ = 0.

Figure 6 shows the evolution of the disk density as a function of the iterations. The variation of the disk density over the last 9000 iterations is: Δ(FVF) = 0.8449–0.8440 < 0.001. For a fixed diameter distribution and *N* = 1000 disks, the disk density FVF varies < 0.001 after 26000 iterations. For these inputs (μ = 3 μm, σ^2^ = 1 μm and Δ = 0) the algorithm can be stopped after 26000 iterations beyond which FVF don't vary anymore with a precision of 0.001.

### Application in the white matter

**Figure 7:**
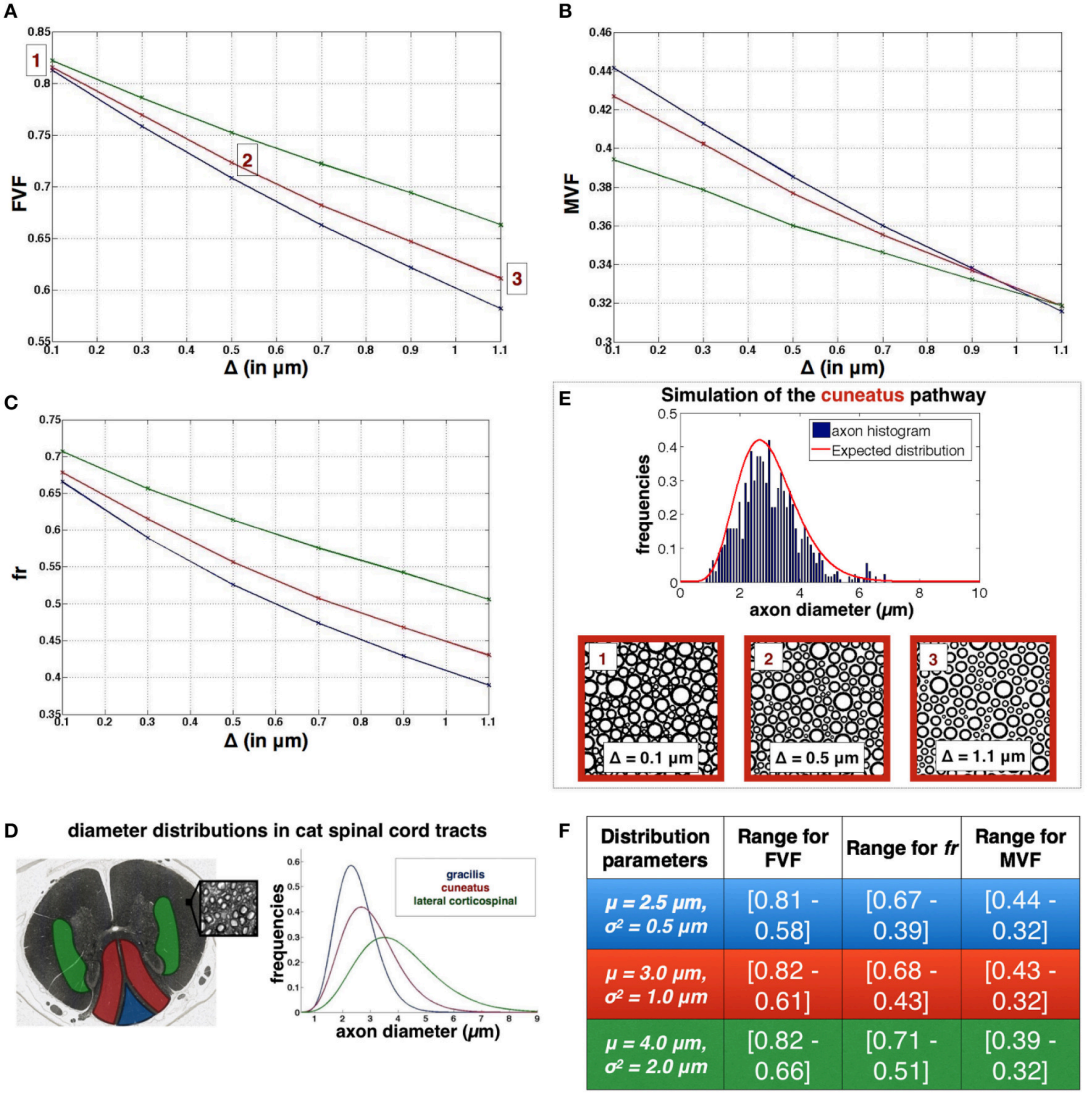
**Application for simulating white matter microstructure (A–C)**. Evolution of FVF, *fr* and MVF when different gaps between axons Δ are applied. **(D)** Definition of the three diameter distributions used in the plots, obtained from three spinal regions of a cat spinal cord (blue: gracilis blue, red:cuneatus, green: lateral corticospinal), adapted with permission from Zaimi et al. (2016). **(E)** Results of axon packing for the cuneatus pathway. Axon diameter histogram and Packing results obtained for three gaps Δ. **(F)** Ranges of values for FVF, *fr* and MVF for the three regions: gracilis (first line), cuneatus (second line), lateral corticospinal (third line).

Figure 7 shows the resulting evolution of axonal density FVF, of the restricted volume fraction *fr* and the (MVF) when the distance between axons (Δ) varied from 0.1 to 1.1 μm, and for three regions of the spinal cord (cuneatus, gracilis and lateral corticospinal) with typical diameter distributions (see Figure 7D). Figures 7A–C shows the range of values for FVF, *fr* and MVF for the three different regions. For each distribution, R_*overlap*_ is lower than 0.005% for Δ = 0.1 μm, null in every other cases. Note that each simulation took 40 min on a iMac i5 3.4 GHz (quad core).

## Discussion

In this paper, a new algorithm for simulating dense packing of disks and particularly adapted to model the white matter is presented. The algorithm correctly converges toward an hexagonal packing in the monodisperse case and provides highly reproducible results. An application of the algorithm, presented in this manuscript, aimed to get the evolution for FVF, *fr*, and MVF as a function of the gap between axons for different distributions found in the spinal cord of a cat. In this section, the performance and the limitations of AxonPacking will be discussed, results presented in Figure 7 will be interpreted, and finally, the pros and cons of using AxonPacking to simulate the white matter will be detailed.

### Validation and convergence of axonpacking

AxonPacking was designed to optimize the density of the disks by migrating them with a constant velocity toward the center. As seen in the results section, the algorithm correctly converges toward the optimal solution in the configuration where all disks are identical: the final solution matches the hexagonal structure that is the theoretically highest reachable density for such a problem. However, in other configurations, such as in a bidisperse (two different diameters) or polydisperse disk packing configuration, no known theoretical solution exists to calculate the highest density, and the solution provided by AxonPacking might not be optimal. Qualitatively however, we observe that only few empty spaces remain in the final solution. Also the final disk density is highly reproducible over runs (see Figure 4), no matter the initialization of the disks on the initial grid, suggesting that the final solution is physically optimal for particle dynamics (MRJ state).

While the proposed algorithm tolerates some disk overlapping, the residual overlapping area is negligible (<0.1% when the gap is null) and it is possible to completely avoid overlap by adding a small gap between disks (set as input parameter of AxonPacking). Such small overlap is obtained by turning off the constant velocity toward the center when overlapping occurs.

Figure 6 shows that the optimal solution, although very stable, oscillates toward an optimal value. This oscillation is attributed to the bouncings between disks, that was minimized by using velocities much smaller than the disks diameter (0.01 μm/iteration for attraction and 0.1 μm/iteration for repulsion vs. diameters >0.2 μm). No stopping criteria was implemented here. Instead, the number of iterations was set to a large value (*N* = 30000), which was experimentally found to be sufficient to obtain a stable solution in our simulations (see one example in Figure 6). Thanks to the good reproducibility of the simulations, we were able to generate a dataset of axon packing for a variety of diameter distributions, gaps Δ and fiber density^5^.

### Using axonpacking for modeling white matter

AxonPacking makes a couple of assumption and simplifications in order to model the white matter. First, the algorithm assumes perfectly circular and non-deformable axons. This choice was motivated by the observation that most of the axons are qualitatively circular on electron microscopy or coherent anti-stokes Raman spectroscopy (Perge et al., 2012; Bégin et al., 2014; Zaimi et al., 2016). However, axons, and more particularly the large ones, can show some deformation with more elliptical or even tortuous shapes. These deformations would allow slightly higher density that the ones found in this work. Future work could consider controlled deformations of the circular shape.

Second, AxonPacking assumes a fixed gap Δ between axons. This gap is necessary to leave enough space for the extracellular matrix and the glial cells. As observed in histological images, there exists a variation of the average gap between axons (Zaimi et al., 2016) that could be attributed to different category and proportion of glial cells between regions (Olude et al., 2015). In this work, the goal was to find the global effect of the gap between axons on metrics *fr*, MVF, and FVF. However, a fixed gap in each regions is a strong assumption that could be refined by considering a gap distribution. Note that a fixed gap could also affect the diffusion models, such as the tortuosity model (Alexander et al., 2010) and the time-dependent diffusion model (Fieremans et al., 2008, 2016; Burcaw et al., 2015).

Third, AxonPacking assumes that axons are packed with maximum density, as suggested by the natural tendency of white matter organization (Perge et al., 2009). This trend might deviate from the true organization across white matter pathways and peripheral nerves, across species and pathologies. In order to adapt the simulation for these different scenarios, user can adapt the gap between axons.

Fourth, AxonPacking considers that axon fibers are parallel. While this assumption doesn't hold in regions that present a fanning or tortuosity of the fibers, this assumption holds in a couple of structures of the central nervous system, such as the spinal cord. This is confirmed in the spinal cord by a consistency of microstructural MRI metrics (axon diameter index, fiber density) along the spinal cord (Duval et al., 2015). However, it is important to stress that even in structures that consist of highly parallel fibers, some dispersion can be observed, as shown in the spinal cord (Grussu et al., 2015) and in the corpus callosum (Budde and Annese, 2013). This is a major issue for modeling approaches, many of which rely on the assumption of perfectly parallel cylinders in a 3D arrangement. When this assumption is not valid, this can have a large impact on the inferred parameters.

Fifth, white matter is not only composed of myelinated axons but also contains blood vessels, nerve cells body, fissures, and lakes containing cerebrospinal fluid. In future work, more realistic representation of the white matter can be included in the packing algorithm.

Sixth, all axons in AxonPacking are myelinated axons. While non-myelinated axons also exist in the white matter (Biedenbach et al., 1986; Lamantia and Rakic, 1990), their fraction is generally small. For example, the proportion of myelinated axons (by raw count) among all axons ranges from 69 to 97% in the monkey corpus callosum (Lamantia and Rakic, 1990). Moreover, the mean diameter of the unmyelinated fibers is much smaller than the myelinated one, respectively, 0.18 and 2.2 μm (Biedenbach et al., 1986), which somewhat minimizes the impact of unmyelinated fibers on the calculation of FVF. Again, future work can address this issue if desired by the modeling study.

Finally, the relationship between g-ratio and axon caliber (Figure 1D) might differ between species and regions, which could impact the values found for FVF, MVF, and *fr*. More advanced relationships that use a probabilistic approach could be considered in future versions of AxonPacking. AxonPacking could be applied to other species exhibiting different microstructure properties, such as monkey (Lamantia and Rakic, 1990), human (Aboitiz et al., 1992), or rat (Leenen et al., 1985).

### Simulating the variation of fr, MVF, and FVF in the white matter

In this study, we chose a range of Δ (distance between the edge of axons) from 0 to 1.1 μm. This range was chosen by measuring the distance between a few axons in a couple of regions of the cat spinal cord (data not shown). Future work will try to draw the probability distribution of the gaps between axons on electron microscopy images. Result section “Application in the white matter” shows how FVF, *fr*, and MVF vary as a function of Δ for different diameter distributions. Several observations can be reported.

First, for a gap Δ = 0, a change in diameter distribution mostly impacts MVF (10% variation in our simulations) and *fr* (~7% variation) with inverse trends. The presence of large axons lowers MVF because large axons have bigger g-ratio and thus proportionally less myelin (see Figure 1D). Thinner myelin leaves space for the intra-axonal water, increasing *fr*. We observe, however, very small impact on FVF (~1%).

Second, we observe a larger sensitivity to the gap Δ for *fr* than MVF. For instance, in the cuneatus tract, *fr*(Δ = 0)−*fr*(Δ = 1.1) = 0.25 while MVF(Δ = 0)−MVF(Δ = 1.1) = 0.11. In addition, *fr* is more sensitive to differences of diameter distribution than MVF. This observation is in agreement with MRI experiments: a larger standard deviation for metric *fr* (0.04) than MVF (0.02) was measured in the human spinal cord white matter (Duval et al., 2017). Note also that values obtained with AxonPacking (Figure 7F) are in agreement with the values obtained with MRI [mean(*fr*) = 0.51, mean(MVF) = 0.27 and mean(FVF) = 0.66]. These values also provide upper bounds for the different metrics (values at Δ = 0).

Third, FVF as a function Δ varies differently depending on axon diameter distribution. Indeed, in regions presenting large axons, a small gap (e.g., Δ < 0.1^*^μ) only has a small impact on the calculated volume fractions. However, when axon diameters are small, a smaller gap will yield a drop of FVF. The same effect is observed on *fr* and MVF curves. As a result, MVF is robust to the mean axon diameter when the ratio Δ/μ is close to 0.3 (the three curves converge). In this regime, MVF is driven mostly by the g-ratio.

Note that the curves reported on Figure 7 can also be used to choose the right gap Δ in order to generate synthetic axonal packing with particular fiber density or (MVF). Also, these results can be used as a lookup table to estimate parameters *fr*, MVF or FVF knowing the diameter distribution and one of the three parameters without running the simulator.

## Conclusion

AxonPacking is a novel open-source software for simulating white matter microstructure, in which axons are assumed to be parallel cylinders. AxonPacking generates random disk packing with user-defined diameter distribution and gaps between the disks, and then computes the following microstructure features: (MVF), (FVF) and restricted water fraction (*fr*). AxonPacking can be useful for validating diffusion models as well as for enabling researchers to study the interplay between microstructure parameters when evaluating qMRI methods. AxonPacking can be downloaded at https://github.com/neuropoly/axonpacking.

## Author contributions

TM and TD designed the study, performed the experiments and wrote the manuscript. NS and JC designed the study and wrote the manuscript.

### Conflict of interest statement

The authors declare that the research was conducted in the absence of any commercial or financial relationships that could be construed as a potential conflict of interest.

## Footnotes

^1^http://cims.nyu.edu/~donev/Packing/PackLSD/Instructions.html

^2^http://camino.cs.ucl.ac.uk/

^3^https://github.com/rfdougherty/dSim

^4^https://github.com/neuropoly/axonpacking.

^5^https://github.com/neuropoly/axonpacking/tree/master/results.
